# Dataset on transcriptome profiling of corneal endothelium from patients with Fuchs endothelial corneal dystrophy

**DOI:** 10.1016/j.dib.2019.104047

**Published:** 2019-05-23

**Authors:** Anastasia S. Nikitina, Alexandra V. Belodedova, Boris E. Malyugin, Elena I. Sharova, Elena S. Kostryukova, Andrey K. Larin, Vladimir A. Veselovsky, Olga P. Antonova, Liubov O. Skorodumova

**Affiliations:** a Federal Research and Clinical Center of Physical-Chemical Medicine of Federal Medical Biological Agency, Moscow, Russia; b Moscow Institute of Physics and Technology, Dolgoprudnyi, Russia; c The S. Fyodorov Eye Microsurgery Federal State Institution, Moscow, Russia

**Keywords:** Fuchs endothelial corneal dystrophy, RNA-Seq, Transcriptome

## Abstract

Fuchs endothelial corneal dystrophy (FECD) is a bilateral inherited eye disease with advanced forms only treatable by corneal transplantation. The pathogenesis of FECD has not been worked out yet, however, trinucleotide repeat polymorphism CTG18.1 in the TCF4 gene has recently been associated with late-onset FECD. Gene expression profiling of corneal endothelium with and without this expansion can help elucidate molecular mechanisms of the disease development. Current data article represents whole transcriptome profiles of corneal endothelium obtained from 12 patients with FECD and 6 control tissues from eye bank donors. RNA sequencing data is available at NCBI Sequence Read Archive under Accession No. PRJNA524323. In addition, each patient and donor were genotyped for CTG18.1 expansion and the corresponding numbers of CTG repeats in the TCF4 gene are provided within this article. The dataset includes samples from FECD patients both with and without CTG18.1 expansion.

**Table:** Specifications table

Subject area	Biology
More specific subject area	Fuchs endothelial corneal dystrophy
Type of data	Text (FASTQ sequence files), table
How data was acquired	High-throughput RNA sequencing using Illumina HiSeq 2500 Sequencing System
Data format	Raw
Experimental factors	Samples of corneal endothelium were obtained from patients with FECD during the endothelial keratoplasty. Control samples of corneal endothelium were collected from donors of the eye bank. Blood samples and iris tissues were also obtained for genotyping from patients and donors respectively.
Experimental features	Total RNA was isolated from samples of corneal endothelium. After removing traces of DNA and ribosomal RNA whole transcriptome libraries were constructed and sequenced. Genotyping for CTG18.1 trinucleotide repeat expansion was performed using short tandem repeat (STR) and triplet primed PCR (TP-PCR) techniques.
Data source location	Moscow, Russia
Data accessibility	Raw data was deposited at NCBI SRA database under accession No. PRJNA524323 https://www.ncbi.nlm.nih.gov/sra/PRJNA524323
Related research article	L.O. Skorodumova, A. V. Belodedova, O.P. Antonova, E.I. Sharova, T.A. Akopian, O. V. Selezneva, E.S. Kostryukova, B.E. Malyugin. CTG18.1 Expansion is the Best Classifier of Late-Onset Fuchs' Corneal Dystrophy Among 10 Biomarkers in a Cohort From the European Part of Russia. Investig. Ophthalmology Vis. Sci. 59 (2018) 4748 [1]

**Table:** 

**Value of the Data** • The dataset represents transcriptomes of samples from FECD patients as well as from control donors which can be compared to reveal molecular pathways altered in corresponding endothelial cells leading to their accelerated death. • The identification of affected metabolic and signaling pathways may indicate the direction of research for the development of specific non-surgical treatment of FECD. • All subjects in this study have already been genotyped for CTG18.1 expansion. The dataset includes samples from patients both with and without the expansion, which can be valuable for identifying the role this genetic variation plays in FECD pathogenesis. • Expression profiles are available in the form of raw sequencing reads that can be further processed by researchers using their own bioinformatic algorithms and analyzed together with their own data.

## 1 Data

The dataset contains raw sequencing data obtained through the transcriptome sequencing of corneal endothelium from 12 patients with FECD and 6 donors. The data files (reads in FASTQ format) were deposited at NCBI SRA database under project accession No. PRJNA524323. Information about tissue samples collected from patients with FECD and control samples from donors is presented in Table 1 and Table 2, respectively.

**Table 1:** Samples of corneal endothelium obtained from patients with FECD.

Sample ID	Age, years	Sex	Diagnosis	FECD stage	Comorbidity	CEC (corneal endothelial cell) density	Time between cataract surgery and endothelial keratoplasty
Dfu_201	70	f	ОD FECD	stage II	Complicated cataract^a^	568	simultaneous
Dfu_202	63	m	ОD FECD	stage II	Complicated cataract	478	simultaneous
Dfu_203	57	f	OS FECD	stage II	Complicated cataract	463	simultaneous
Dfu_205	64	f	OS FECD	stage II	Complicated cataract	463	simultaneous
Dfu_207	79	f	OS FECD	stage II	Complicated cataract	486	simultaneous
Dfu_209	56	f	ОD FECD	stage IV	Neovascularization, Pseudophakia	–	22 months
Dfu_210	64	m	ОD FECD	stage II	Complicated cataract	489	simultaneous
Dfu_212	72	f	OS FECD	stage III	Pseudophakia	501	13 months
Dfu_213	74	m	OS FECD	stage II	Complicated cataract	465	simultaneous
Dfu_215	70	f	ОD FECD	stage II	Complicated cataract	464	simultaneous
Dfu_217	57	f	ОD FECD	stage III	Complicated cataract	503	simultaneous
Dfu_219	69	f	OS FECD	stage II	Complicated cataract	512	simultaneous

a The cataract was classified as complicated if the patient had any other concomitant eye disease, which was FECD for all patients from this sample group.

**Table 2:** Control samples of corneal endothelium obtained from donors.

Sample ID	Age, years	Sex	Cause of death	CEC density
С_201	47	f	Cardiomyopathy	2976
C_203	63	m	Asphyxia	2864
C_205	54	f	Acute cardiovascular insufficiency	2924
C_206	65	f	Acute cardiovascular insufficiency	2931
C_207	64	f	Acute cardiovascular insufficiency	2789
C_208	61	m	Acute cardiovascular insufficiency	2812

All patients and donors were also characterized by genotyping for trinucleotide repeat polymorphism CTG18.1 in the TCF4 gene (Table 3).

**Table 3:** The data on genotyping patients with FECD and donors for CTG18.1 expansion.

Sample ID	Sample group	TCF4 expansion status	Number of expanded TCF4 alleles	Number of repeats in smaller TCF4 allele	Number of repeats in larger TCF4 allele
Dfu_201	FECD	expanded	1	12	156
Dfu_202	FECD	expanded	2	72	72
Dfu_203	FECD	expanded	2	72	72
Dfu_205	FECD	expanded	1	32	127
Dfu_207	FECD	expanded	1	11	87
Dfu_209	FECD	non-expanded	0	11	14
Dfu_210	FECD	non-expanded	0	11	14
Dfu_212	FECD	expanded	1	14	44
Dfu_213	FECD	expanded	1	14	104
Dfu_215	FECD	expanded	1	21	114
Dfu_217	FECD	non-expanded	0	14	17
Dfu_219	FECD	non-expanded	0	15	17
C_201	Control	non-expanded	0	14	18
C_203	Control	non-expanded	0	11	18
C_205	Control	non-expanded	0	11	17
C_206	Control	non-expanded	0	11	15
C_207	Control	non-expanded	0	11	17
C_208	Control	non-expanded	0	17	17

## 2 Experimental design, materials, and methods

### 2.1 Ethical statements

This study was approved by the Institutional Review Board of The S. Fyodorov Eye Microsurgery Federal State Institution and was carried out in accordance with The Code of Ethics of the World Medical Association (Declaration of Helsinki).

### 2.2 Collection of tissue and blood samples from FECD patients

Samples of corneal endothelium were obtained from patients diagnosed with FECD and undergoing corneal transplantation at The S. Fyodorov Eye Microsurgery Federal State Institution. All patients included in this study had signed an informed consent form. Table 2 summarizes information about tissue samples collected from patients with FECD. Disease stage was identified according to the Volkov and Dronov classification as described in Ref. [1].

During the endothelial keratoplasty procedure, circular descemetorhexis with a diameter of 7–8 mm was performed using a special hook to obtain samples of corneal endothelium/Descemet's membrane (CEC-DM) complex. RNA in CEC-DM complex was stabilized using RNAlater solution (ThermoFisher Scientific, USA) according to the manufacturer's instructions. Venous blood (4–6 mL) was collected from each patient into vacutainer tubes with EDTA (Becton Dickinson, USA). Blood samples were stored at −20 °C prior to DNA extraction.

### 2.3 Collection of control tissue samples from donors

Control samples of corneal endothelium were collected at the Eye Bank of The S. Fyodorov Eye Microsurgery Federal State Institution. Donor samples were selected from those not suitable for corneal transplantation. Information about control tissue samples collected from donors is presented in Table 3.

The eyeball was stored for a maximum of 1 day before extraction of the corneoscleral disc which then was kept in a preservative medium for the period of up to 12 hours. The preservative medium contained: 25% of medium 199, 25% of F-10 medium, 45.3% of DMEM, 2% dextran, 2.7% chondroitin sulfate, gentamicin sulfate 0.00014%, amphotericin B 0.00015%. Afterward, the corneoscleral disc was placed in an artificial anterior chamber endothelial side up. Circular excision of CEC-DM complex 7–8 mm in diameter was performed using spatula and tweezers. After isolation, the donor CEC-DM complexes were immediately immersed in the RNAlater solution (ThermoFisher Scientific, USA). Iris samples were also collected from donors for the TCF4 repeats expansion genotyping.

### 2.4 CEC density measurement

The density of corneal endothelial cells was measured using Tomey EM-3000 Specular Microscope (Tomey, USA). The shooting method was non-contact, the measurement mode was manual/automatic, the shooting area was 0.25 mm × 0.54 mm with 7 capture points (central + 6 points on the periphery). The accuracy of corneal thickness measurement was ±10nm ". CEC density was evaluated if at least one of the capture points had enough transparency for the analysis.

### 2.5 DNA extraction

DNA was isolated from thawed blood samples with Gentra Puregene Blood Kit (Qiagen, Germany) according to the manufacturer's protocol. DNA was resuspended in a low TE buffer to a final concentration of 10 ng/μl. DNA from iris samples was extracted using ZR Genomic DNA Tissue MiniPrep (Zymo Research, USA).

### 2.6 Genotyping

Identification of the number of CTG repeats within the CTG18.1 allele in the TCF4 gene was carried out using short tandem repeat (STR) and triplet primed PCR (TP-PCR) techniques exactly according to the procedure described in Ref. [1]. The TCF4 allele was classified as expanded if the number of CTG repeats was ≥40 according to previously reported literature [2], [3].

### 2.7 RNA extraction

Disruption and homogenization of tissue samples were performed with TissueRuptor II (Qiagen, Germany). The time of homogenization was 20 seconds. Total RNA was isolated using RNeasy Micro Kit (Qiagen) following the manufacturer's protocol. Traces of DNA were removed with TURBO DNA-free Kit (ThermoFisher Scientific). RNA concentration was assessed using the Qubit 2 instrument (Invitrogen, USA) with Qubit HS RNA Assay Kit (ThermoFisher Scientific, USA). The quality of total RNA expressed as RNA Integrity Number (RIN) was determined with Bioanalyzer 2100 instrument (Agilent, USA) using an Agilent RNA Pico 6000 Kit (Agilent, USA) [4]. On average, the RIN value of 6.5 was obtained, which is typical for RNA samples isolated from tissues [5].

### 2.8 Transcriptome library preparation and sequencing

Ribosomal RNA was depleted using NebNext rRNA Depletion Kit (Human/Mouse/Rat) (New England Biolabs, USA). Transcriptome libraries were constructed with NEBNext Ultra II Directional Library Prep Kit for Illumina (New England Biolabs, USA) and Multiplex Oligos for Illumina (96 Indexes) (New England Biolabs, USA). The resulting paired-end libraries were sequenced on the Illumina HiSeq 2500 instrument with 2 × 125 cycles using HiSeq SBS Kit v4 (Illumina, USA).
