# Supplementary material for: Dataset on transcriptome profiling of corneal endothelium from patients with Fuchs endothelial corneal dystrophy
Source: Data Brief. 2019 May 23;25:104047. doi: 10.1016/j.dib.2019.104047 (PMC6558234; doi:10.1016/j.dib.2019.104047)
Supplement: Multimedia component 1 [file mmc1.docx]

The authors declare no conflict of interests for this article.
